# Antimicrobial Peptides Induce Cell Death in Marginal Zone Lymphoma Models Resistant to Targeted Therapies

**DOI:** 10.1002/jha2.70267

**Published:** 2026-03-30

**Authors:** Filippo Spriano, Alberto J. Arribas, Fangwen Zhang, Elisa Civanelli, Maria Luisa Mangoni, Francesco Buonocore, Francesco Bertoni

**Affiliations:** ^1^ Faculty of Biomedical Sciences Institute of Oncology Research (IOR) USI Bellinzona Switzerland; ^2^ Laboratory Affiliated to Pasteur Italia‐Fondazione Cenci Bolognetti Department of Biochemical Sciences Sapienza University of Rome Rome Italy; ^3^ Department for Innovation in Biological Agro‐Food and Forest Systems University of Tuscia Viterbo Italy; ^4^ Oncology Institute of Southern Switzerland (IOSI) Ente Ospedaliero Cantonale Bellinzona Switzerland

**Keywords:** BTK, chionodracines, lymphoma, peptides, resistance, temporins, trematocines

## Abstract

**Introduction:**

Marginal zone lymphoma (MZL) is an indolent yet incurable B‐cell malignancy in which targeted agents such as BTK and PI3K inhibitors frequently fail due to resistance or toxicity. Antimicrobial peptides (AMPs), evolutionarily conserved effectors of innate immunity, possess selective cytotoxicity against malignant cells by exploiting tumor‐specific membrane alterations.

**Methods:**

Peptides were synthesized and tested for their anti‐proliferative activity in MZL cell lines.

**Results:**

We evaluated the antitumor activity of seven natural AMPs, including Antarctic fish–derived trematocines and chionodracine variants, and amphibian temporins, against MZL cell lines (VL51, Karpas1718) and derivatives resistant to BTK, PI3Kδ, or PI3Kα/δ inhibitors. Among them, W‐trematocine and temporin L demonstrated potent dose‐dependent cytotoxicity with IC_50_ values of 5.7–10 µM, maintaining full activity in all resistant models. Other peptides showed moderate activity, while chionodracine‐1 was inactive. Notably, W‐trematocine displayed minimal toxicity toward nonmalignant cells in prior studies, underscoring its selectivity. AMP‐mediated killing, driven by membrane disruption and non‐apoptotic death pathways, bypassed conventional resistance mechanisms, suggesting therapeutic potential in relapsed/refractory disease.

**Conclusion:**

Our findings highlight natural AMPs as promising candidates for development in drug‐resistant MZL, warranting further optimization and preclinical validation.

1

Marginal zone lymphoma (MZL) is a heterogeneous group of indolent B‐cell non‐Hodgkin lymphomas that includes extranodal mucosa‐associated lymphoid tissue (MALT) lymphoma, nodal MZL, and splenic MZL [1, 2]. Although these lymphomas are generally slow‐growing, they remain incurable in most patients. In recent years, targeted therapies have substantially improved clinical outcomes. Agents such as Bruton's tyrosine kinase (BTK) inhibitors (ibrutinib, zanubrutinib) and phosphoinositide 3‐kinase (PI3K) inhibitors (idelalisib, copanlisib) have become central to the therapeutic landscape [2]. However, their efficacy is often limited by resistance mutations, activation of alternative signaling pathways, or intolerable toxicities that necessitate treatment discontinuation [2]. Therefore, there is an unmet clinical need to identify novel therapeutic strategies that bypass traditional resistance mechanisms.

Antimicrobial peptides (AMPs) are evolutionarily conserved bioactive molecules that represent one of the most ancient and fundamental strategies of host defense against microbial invasion [3]. These small peptides (typically 5–50 amino acid residues in their biologically active sequence) are widely distributed across all forms of life, from bacteria and fungi to plants, invertebrates, and vertebrates. Their broad presence underscores a critical role in innate immunity, where they not only function as microbicidal agents but also modulate the immune response, promote wound healing, and regulate inflammation. AMPs primarily exert their biological activity by interacting with and disrupting microbial membranes; their amphipathic conformation, coupled with a net positive charge, allows preferential binding to the negatively charged phospholipids in bacterial cell walls and fungal membranes [4]. While AMPs have long been studied for their antimicrobial properties, there is now increasing attention to their ability to induce cytotoxicity in malignant cells with minimal effects on healthy tissues [5]. The rationale for this lies in the similarities between microbial and tumor cell membranes. Neoplastic cells, particularly those undergoing rapid proliferation, frequently carry a net negative charge due to exposure of phosphatidylserine, altered glycosylation patterns, and increased heparan sulfate proteoglycans [6]. These changes provide a biochemical basis for a selective targeting of cancer cells by cationic AMPs, while sparing most normal cells. AMPs generally compromise plasma membrane integrity, induce mitochondrial dysfunction, or trigger non‐apoptotic forms of cell death such as necrosis [5, 7]. These unique mechanisms raise the possibility that AMPs could be particularly useful in drug‐resistant cancers, where canonical apoptotic pathways are impaired.

We investigated the potential of natural AMPs to exert cytotoxic activity against MZL cell lines, including variants resistant to currently available targeted agents. Seven peptides of natural origin were selected based on their previously reported antimicrobial or antitumor properties. These included three peptides from Antarctic fish, two variants of trematocine derived from *Trematomus bernacchii*, and one variant of a peptide, chionodracine, isolated from *Chionodraco hamatus*, as well as four temporins from amphibians (*Rana temporaria*) [8, 9, 10, 11] (Figure 1A). The Antarctic peptides are of particular interest because organisms inhabiting extreme environments often evolve structurally unique peptides with enhanced stability and bioactivity [8, 9]. Temporins, in turn, are among the smallest AMPs and have been extensively studied for their amphipathic α‐helical conformations, which favor interactions with membranes [7].

**FIGURE 1:**
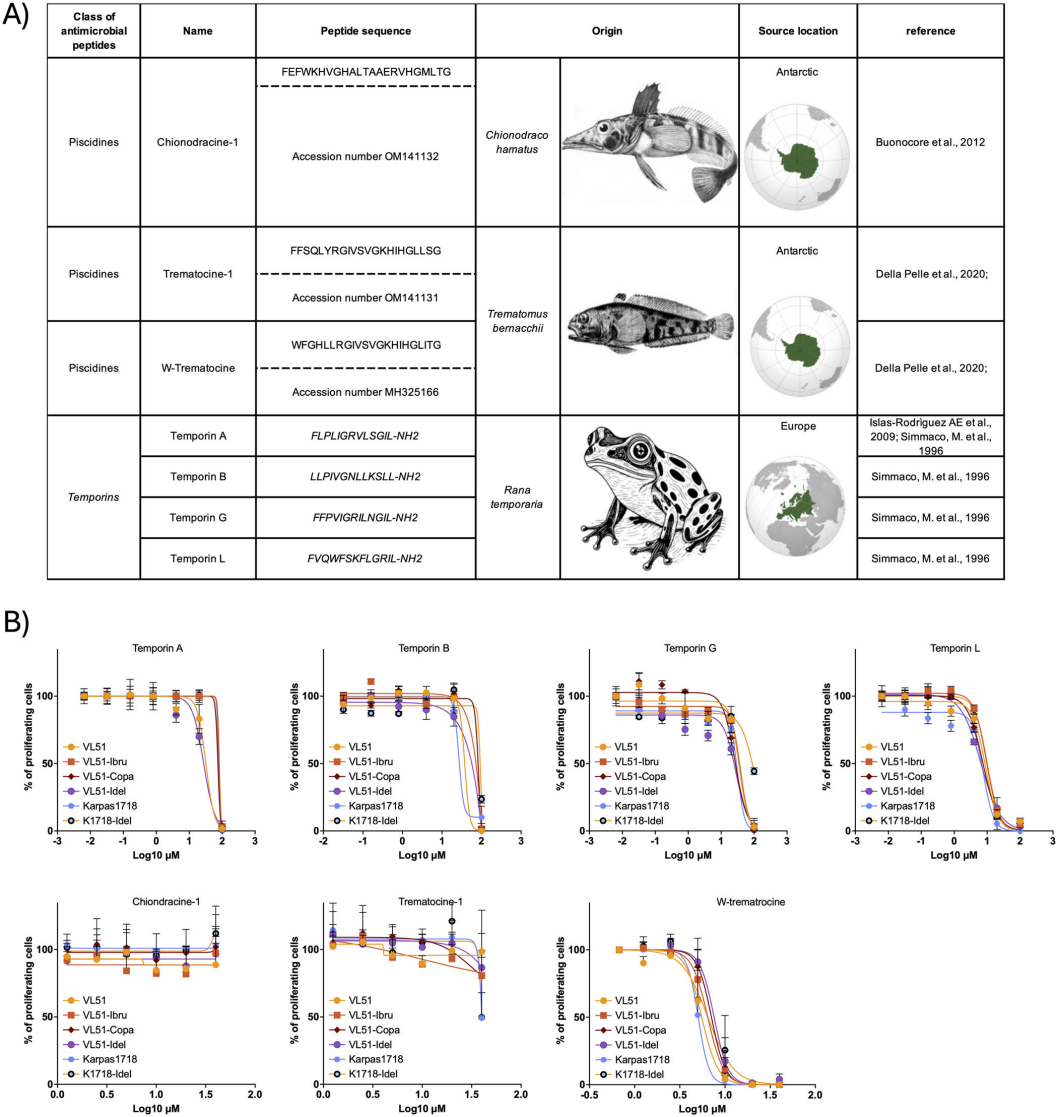
Antimicrobial peptides (AMPs), their species of origin, and effects on lymphoma cell lines. (A) Peptides derived from *Chionodraco hamatus* (chionodracine peptide) [8]*, Trematomus bernacchii* (trematocine peptides) [9], and *Rana temporaria* (temporin peptides) [10, 11] were tested for their potential antitumor activity. The geographic distribution of each species is highlighted on the maps. (B) Dose–response curves of peptides were generated in parental and drug‐resistant (idelalisib, ibrutinib, copanlisib) VL51 cells and in parental and idelalisib‐resistant Karpas1718 cells. *Y*‐axis: Percentage of proliferating cells relative to control; *X*‐axis: Compound concentrations (µM, Log10 scale). Average of two independent biological replicates. Methods are described in the Supporting Information.

For in vitro testing, we used two human MZL cell lines, VL51 and Karpas1718, along with derivative clones rendered resistant by prolonged exposure to the BTK inhibitor ibrutinib, the PI3Kδ inhibitor idelalisib, or the PI3Kα/δ inhibitor copanlisib (Table S1; methods described in the Supporting Information). Our results revealed a differential spectrum of activity among the tested AMPs. Chionodracine‐1 showed no measurable cytotoxic effect at concentrations up to 50 µM. In contrast, W‐trematocine, a tryptophan‐enriched variant of trematocine, and temporin L exhibited pronounced, dose‐dependent cytotoxicity in both parental and resistant VL51 and Karpas1718 cells (Figure 1B; Table S2). The calculated IC_50_ values ranged from 5 to 10 µM (Table S2). Notably, cytotoxicity was fully preserved in all resistant cell line derivatives, suggesting that the mechanism of action of these peptides bypasses the canonical signaling pathways targeted by BTK, PI3K, and BCL2 inhibitors. Additional peptides, including trematocine‐1 and other temporin variants, showed moderate cytotoxicity, with IC_50_ values of 25–50 µM (Figure 1B), suggesting a potential structure–activity relationship that merits further optimization. Importantly, earlier work showed that exposure of primary human fibroblasts and rabbit erythrocytes to W‐trematocine resulted in negligible cytotoxicity (up to 25 µM, 24 h) and minimal hemolysis, respectively [9]. These findings were validated by treating lymphoma cells and peripheral blood mononuclear cells (PBMCs) in parallel. W‐trematocine and temporin L (10 µM) exhibited potent anti‐lymphoma activity as early as 1 h after exposure, whereas PBMCs were markedly less sensitive to the treatment (Figure S1A). Treated lymphoma cells showed morphological alterations and concomitant Annexin V and PI positivity, indicating early loss of plasma membrane integrity rather than canonical early apoptosis (Figure S1B), similar to what was observed in bacteria exposed to W‐trematocine [12]. These results underscore the distinct mode of action of AMPs compared with conventional small‐molecule inhibitors. While acquired resistance to BTK or PI3K inhibitors often involves secondary mutations in the drug‐binding site, activation of alternative kinases, or evasion of apoptosis through upregulation of anti‐apoptotic proteins, these adaptations are unlikely to protect tumor cells from AMP‐mediated membrane disruption. In addition, the preserved activity of AMPs in resistant cell lines suggests that these peptides could serve as salvage therapies for relapsed/refractory MZL, either as monotherapy or in rational combinations.

In conclusion, our study demonstrates that natural AMPs, particularly W‐trematocine and temporin L, exert potent antitumor effects against MZL cells, including variants resistant to BTK, PI3K, and BCL2 inhibitors. These findings position AMPs as promising candidates for the development of novel therapeutics for drug‐refractory B‐cell malignancies. Future studies will focus on elucidating the precise mechanisms of AMP‐induced cell death and on evaluating selectivity for malignant versus normal cells. Optimizing peptide stability and delivery will be the critical next steps toward clinical translation. Given their unique mode of action and preserved activity in resistant disease, AMPs may ultimately complement or expand the current therapeutic.

## Author Contributions

**Filippo Spriano**: performed experiments, interpreted data, and co‐wrote the manuscript. **Alberto J. Arribas**: performed experiments and interpreted data. **Fangwen Zhang**: performed experiments. **Elisa Civanelli**: performed experiments. **Maria Luisa Mangoni**: co‐designed the study, provided reagents, interpreted data, and provided advice. **Francesco Buonocore**: co‐designed the study, provided reagents, interpreted data, and provided advice. **Francesco Bertoni**: co‐designed the study, interpreted data, supervised the study, and co‐wrote the manuscript. All authors reviewed and accepted the final version of the manuscript.

## Funding

This work was partially supported by institutional research funds from the Swiss National Science Foundation (SNSF 31003A_163232/1) and the Swiss Cancer Research (KFS‐4727‐02‐2019). F.Z. was supported by the China Scholarship Council (CSC) and the Swiss State Secretariat for Education, Research and Innovation (SERI).

## Ethics Statement

The authors have nothing to report.

## Consent

The authors have nothing to report.

## Conflicts of Interest

Alberto J. Arribas: Travel grant from AstraZeneca, consultant fee for PentixaPharm. Francesco Bertoni: Institutional research funds from BeiGene, Floratek Pharma, Helsinn, HTG Molecular Diagnostics, Ideogen AG, Idorsia Pharmaceuticals Ltd., Immagene, ImmunoGen, iOnctura, Mabtree, Menarini Ricerche, Nordic Nanovector ASA, Oncternal Therapeutics, Spexis AG; consultancy fee from BIMINI Biotech, Floratek Pharma, Helsinn, Immagene, Menarini, Vrise Therapeutics; advisory board fees to institution from Novartis; travel grants from Amgen, AstraZeneca, iOnctura. The other authors declare no conflicts of interest.

## Supporting information




**Supporting File 1**: jha270267‐sup‐0001‐SuppMat.pdf

## Data Availability

All data are available in the main text, in the Supporting Information, or upon request to the authors.
