# Supplementary material for: Antimicrobial Peptides Induce Cell Death in Marginal Zone Lymphoma Models Resistant to Targeted Therapies
Source: EJHaem. 2026 Mar 30;7(2):e70267. doi: 10.1002/jha2.70267 (PMC13239830; doi:10.1002/jha2.70267)
Supplement: Supplementary file 1 — Supporting File 1: jha270267‐sup‐0001‐SuppMat.pdf [file JHA2-7-e70267-s001.pdf]

Antimicrobial Peptides Induce Cell Death in Marginal Zone Lymphoma Models Resistant to Targeted Therapies

Filippo Spriano [1], Alberto J. Arribas [1], Fangwen Zhang [1], Elisa Civanelli [1], Maria Luisa Mangoni [2], Francesco Buonocore [3], Francesco Bertoni [1,4]

[1] Institute of Oncology Research (IOR), Faculty of Biomedical Sciences, USI, Bellinzona, Switzerland; [2] Laboratory Affiliated to Pasteur Italia-Fondazione Cenci Bolognetti, Department of Biochemical Sciences, Sapienza University of Rome, Rome, Italy; [3] Department for Innovation in Biological, Agro-Food and Forest Systems, University of Tuscia, Viterbo, Italy; [4] Oncology Institute of Southern Switzerland (IOSI), Ente Ospedaliero Cantonale, Bellinzona, Switzerland.

Supplementary Methods

Cell lines

The established human cell lines VL51 [1] and Karpas1718 [2], derived from bona fide marginal zone lymphomas (MZL), and their derivatives with secondary resistance obtained by long exposure to the BTK inhibitor ibrutinib, the PI3Kδ inhibitor idelalisib or the PI3Kα/δ inhibitor copanlisib (three from VL51 [3-5], and one from Karpas1718 [6]). Cell lines were cultured in RPMI supplemented with fetal bovine serum (FBS) (10%) and penicillin-streptomycin-neomycin (≈5,000 units penicillin, 5 mg streptomycin, and 10 mg neomycin/mL; Sigma-Aldrich, Darmstadt, Germany). Cell line identities were confirmed by periodic short tandem repeat (STR) DNA fingerprinting using the Promega GenePrint 10 System kit (B9510). Cells were regularly tested for mycoplasma negativity using the MycoAlert Mycoplasma Detection Kit (Lonza, Visp, Switzerland).

Peptides

Temporins were obtained from Biomatik (Wilmington, DE, USA) and synthesized using solid-phase Fmoc chemistry. Purity (>95%) was confirmed by reverse-phase high-performance liquid chromatography (RP-HPLC) while molecular masses were verified by mass spectrometry. Peptides were dissolved in nuclease-free water, and 2 mM stock solutions were prepared [7].
Antarctic peptides were synthesized by Caslo Aps (Caslo Aps Kongens, Lyngby, Denmark) with 98% purity. Purity was confirmed by reverse-phase high-performance liquid chromatography (RP-HPLC), and molecular masses were verified by mass spectrometry. As previously reported, the peptide stock concentration in nuclease-free water (1 mM) was determined spectrophotometrically.

In vitro cytotoxic activity

Cells were manually seeded into 96-well plates (VL51, 10’000 cells per well; SSK41, 20’000 cells per well; Karpas1718, 30’000 cells per well). Treatments were applied manually, and cell viability was assessed after 72 hours using 3-(4,5-dimethylthiazol-2-yl)-2,5-diphenyltetrazolium bromide (MTT). After a 4-hour incubation, the reaction was terminated by adding sodium dodecyl sulfate (SDS) lysis buffer. Plates were analyzed with Cytation3, and IC50s were calculated using the 4-parameter logistic model (R environment or GraphPad Prism, Version 10.6.1). Antarctic peptides were tested at 40µM, with 1:2 dilutions down to 625 nM. Temporins were tested at 100µM, with 1:5 dilutions down to 32 nM.

Analysis of cell death and membrane integrity by Annexin V/Propidium Iodide staining

Cells (Karpas1718-Idel and PBMCs) were manually seeded at 5 × 10^^{5} cells/ml and treated with Temporin-L or W-Trematocine at 10 µM and compared to untreated cells for 1 and 2 hours. Subsequently, cells were stained with Annexin V and propidium iodide (PI) (Annexin V FITC Apoptosis Detection Kit, BMS500FI-300, Life Technologies) following the manufacturer’s instructions and analyzed through flow cytometry and immunofluorescence (Leica Thunder microscope).

References

1. Inokuchi K, Abo J, Takahashi H, Miyake K, Inokuchi S, Dan K, et al. Establishment and characterization of a villous lymphoma cell line from splenic b-cell lymphoma. Leuk. Res. 1995; 19:817-22.
2. Martinez-Climent JA, Sanchez-Izquierdo D, Sarsotti E, Blesa D, Benet I, Climent J, et al. Genomic abnormalities acquired in the blastic transformation of splenic marginal zone b-cell lymphoma. Leuk. Lymphoma 2003; 44:459-64.
3. Arribas AJ, Napoli S, Cascione L, Sartori G, Barnabei L, Gaudio E, et al. Resistance to pi3kdelta inhibitors in marginal zone lymphoma can be reverted by targeting the il-6/pdgfra axis. Haematologica 2022; 107:2685-2697.
4. Arribas A, Napoli S, Cascione L, Gaudio E, Bordone-Pittau R, Barreca M, et al. Secondary resistance to the pi3k inhibitor copanlisib in marginal zone lymphoma. Eur. J. Cancer 2020; 138:S40-S40.
5. Arribas AJ, Guidetti F, Cannas E, Cascione L, Napoli S, Sartori G, et al. Il-16 production is a mechanism of resistance to btk inhibitors and r-chop in lymphomas. bioRxiv 2025; 2025.05.07.652612.
6. Arribas AJ, Napoli S, Cascione L, Barnabei L, Sartori G, Cannas E, et al. Erbb4-mediated signaling is a mediator of resistance to pi3k and btk inhibitors in b-cell lymphoid neoplasms. Mol. Cancer Ther. 2024; 23:368-380.
7. Marcocci ME, Jackowska BG, Prezioso C, Protto V, De Angelis M, Di Leva FS, et al. The inhibition of DNA viruses by the amphibian antimicrobial peptide temporin g: A virological study addressing hsv-1 and jpcyv. Int J Mol Sci 2022; 23:
8. Della Pelle G, Pera G, Belardinelli MC, Gerdol M, Felli M, Crognale S, et al. Trematocine, a novel antimicrobial peptide from the antarctic fish trematomus bernacchii: Identification and biological activity. Antibiotics (Basel) 2020; 9:

Supplementary Figure 1. W-trematocine and Temporin L are active in lymphoma cells but not in PBMCs. Flow cytometric (A) and immunofluorescence (B) analyses of Annexin V/PI staining in Karpas1718-Idel cells and PBMCs after 1- and 2-hour treatments with W-trematocine or Temporin L at 10 µM. (B) Representative images (100x magnification) show brightfield, Annexin V (green), and PI (yellow) staining.

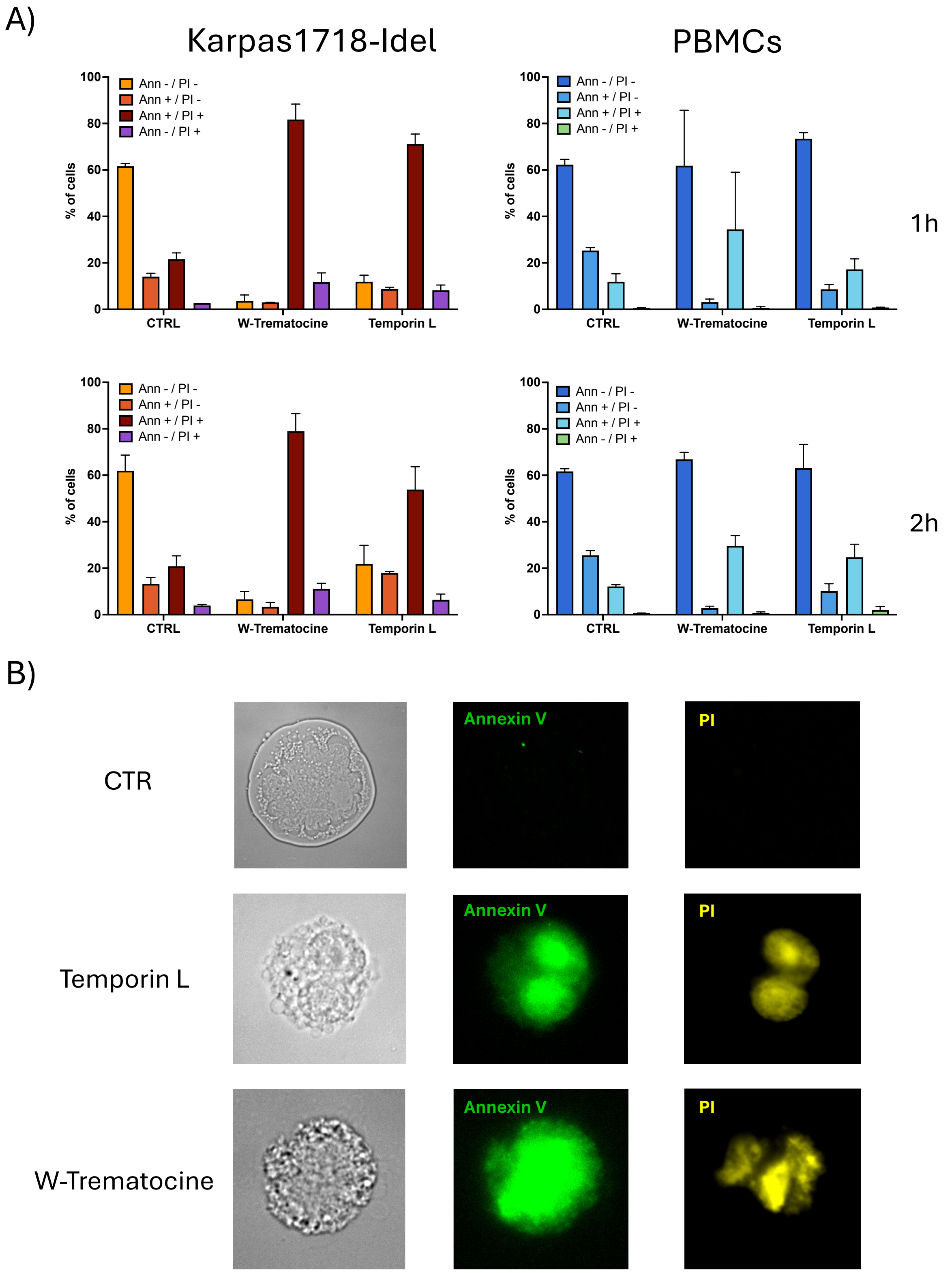

Supplementary Tables

Supplementary Table S1. MZL models used in the work.

Mode	Exposure to	Sensitivity to BTK inhibitors	Sensitivity to BCL2 inhibitors	Sensitivity to PI3K inhibitors	References
VL51	-	yes	yes	yes	[1]
Karpas1718	-	yes	yes	yes	[2]
VL51	Idelalisib	yes	reduced	no	[3]
VL51	Copanlisib	yes	no	no	[4]
VL51	Ibrutinib	no	yes	reduced	[5]
Karpas1718	Idelalisib	no	no	no	[6]

Supplementary Table S2. IC50 values obtained by exposing MZL cells for 72 hours to seven antimicrobial peptides. CI, 95% confidence interval.

	**Chionodracine**		**Trematocine-1**		**W-Trematocine**		**Temporin A**		**Temporin B**		**Temporin G**		**Temporin L**	
**Cell lines**	IC50 (µM)	CI	IC50 (µM)	CI	IC50 (µM)	CI	IC50 (µM)	CI	IC50 (µM)	CI	IC50 (µM)	CI	IC50 (µM)	CI
VL51	*>40*	*N.A.*	*>40*	*N.A.*	*5.5*	*5.1 / 6.2*	*32.0*	*N.A.*	*38.2*	*N.A.*	*35.2*	*N.A.*	*8.9*	*N.A.*
Karpas1718	*>40*	*N.A.*	*>40*	*N.A.*	*5*	*5 / 5.2*	*47.4*	*N.A.*	*44.1*	*N.A.*	*31.0*	*N.A.*	*7.5*	*N.A.*
VL51-Idel	*>40*	*N.A.*	*>40*	*N.A.*	*7.6*	*7.0 / 8.3*	*27.5*	*N.A.*	*31.6*	*N.A.*	*28.2*	*N.A.*	*6.4*	*5.3 / 7.9*
Karpas1718-Idel	*>40*	*N.A.*	*>40*	*N.A.*	*6.5*	*5.3 / 8*	*49.0*	*N.A.*	*71.4*	*N.A.*	*101.3*	*N.A.*	*10.1*	*8.3 / 12.6*
VL51-Copa	*>40*	*N.A.*	*>40*	*N.A.*	*7.1*	*6.9 / 7.3*	*48.8*	*N.A.*	*47.7*	*N.A.*	*26.5*	*N.A.*	*7.4*	*6.6 / 8.5*
VL51-Ibru	*>40*	*N.A.*	*>40*	*N.A.*	*6.5*	*6.3 / 6.6*	*48.5*	*N.A.*	*45.4*	*N.A.*	*38.1*	*N.A.*	*9.6*	*7.6 / 12.7*
